# Marriage survival in new married couples: A competing risks survival analysis

**DOI:** 10.1371/journal.pone.0272908

**Published:** 2022-08-17

**Authors:** Solmaz Norouzi, Roghayeh Tamiz, Sahar Naghizadeh, Khorshid Mobasseri, Leila Imani, Paria Esmaeili, Raha Hamitalab, Fardin Rahmani, Mohammad Asghari Jafarabadi

**Affiliations:** 1 Faculty of Medical Sciences, Department of Biostatistics, Tarbiat Modares University, Tehran, Iran; 2 Department of Biostatistics and Epidemiology, School of Medicine, Zanjan University of Medical Sciences, Zanjan, Iran; 3 Ph.D. in Sociology of Social Groups, Managing Director of Positive Life Social Welfare Clinic, Tabriz, Iran; 4 Department of Biostatistics and Epidemiology, Faculty of Health, Tabriz University of Medical Sciences, Tabriz, Iran; 5 Department of Health Education and Promotion, Faculty of Health, Tabriz University of Medical Sciences, Tabriz, Iran; 6 Road Traffic Injury Research Center, Tabriz University of Medical Sciences, Tabriz, Iran; 7 Cabrini Research, Cabrini Health, Malvern, VIC, Australia; 8 School of Public Health and Preventative Medicine, Faculty of Medicine, Nursing and Health Sciences, Monash University, Monash, VIC, Australia; Polytechnic Institute of Coimbra: Instituto Politecnico de Coimbra, PORTUGAL

## Abstract

**Background and aim:**

Marriage is one of the most important phenomena in human life. The survival of the marriage and the impact of various competing factors on the survival is of high importance. This study aimed at utilizing competing risks survival analysis to investigate the marriage survival of new couples in Tabriz.

**Methods:**

In this longitudinal study, a number of 386 individuals who were married and divorced from 1991 to 2017, were selected by random sampling. The registered information was attained from the general registry office of Tabriz. Data analysis was carried out using the Lunn-McNeil procedure and the results were presented using an adjusted hazard ratio (AHR).

**Results:**

The average age of marriage was about 23.9 (SD 6.6) years. The results of multivariate Lunn-McNeil models indicated that for the competing cause of having a relationship with another person (AHRs range: 1.12 to 2.03), the traditional mode of being familiar (AHRs range: 1.55 to 3.39), family weak role in choosing a wife/spouse (AHRs range: 0.25 to 3.25) and the role of moral-religious commitment (AHRs range: 0.37 to 0.47), along with other causes severed the risk of marriage survival reduction.

**Conclusion:**

According to the results of this study in assessing competing risks, we conclude that the decline in marriage survival is a multifactorial phenomenon. Examining the survival of marriage in order to better understand all the dimensions and factors affecting this phenomenon and providing information to counselors and officials can play an important role in increasing marriage survival.

## Introduction

Marriage is a legitimate way of uniting man and woman in a legal union as a spouse [1]. Marriage helps in reducing chronic illnesses such as heart attack and increasing a sense of happiness, and psychological and physical well-being [2]. However the duration of marriage is not always desirable and in some situations, husband and wife are almost impossible to live which each other, and divorce is the best solution rather than live [3].

Divorce rates in Europe and the United States have increased markedly during the past half century and have stabilized at high levels in 2018, the divorce rate was 2.9 per thousand people in the United States [4]. In England and Wales, the number of divorces increased from approximately 24,000 to about 111,000 per year between 1960 and 2014 [5]. Iran is one of the first seven countries worldwide in terms of divorce [6].

Divorce causes financial, emotional, physical, and legal challenges in couples [7]. The duration of marriage among people varies according to different factors such as the age at marriage, educational level, and employment status [8]. Various studies have assessed the effect of these factors. Some of these factors include sexual incompatibility, lack of intimacy, and lack of communication, marital infidelity, and financial problems influencing divorce [9–13]. The decrease in duration of marriage is worrying and appropriate attention is needed [8]. In overcoming this situation, finding the risk factors of marriage survival would be beneficial for better education of new couples and planning.

On the other hand, Cox regression and parametric models are usually used for situations where the event occurs by one cause because the Cox model by censoring other factors affecting the survival of marriage, Leads to biased estimates, however, these models would not suffice in situations where the event occurs by more than one cause, which are called competing risks. A competing risk is an event that either prevents the observation of the event or modifies the chance that this event occurs. For example, in studies of Decreased marital survival, several causes of decrease have consistently been found, only one of which is the real cause of Decreased marital survival. Decreased marital survival may occur due to X or other causes. This raises the question, what is the leading cause of Decreased in these couples?

In such analyses, competing risks may form an important problem, and should be taken into account in the modeling strategy [14]. The objective of this study was to identify potential determinants with the competing risks approach. To be more specific, the impact of causes such as moral-religious commitment, how to get familiar with the spouse (traditional or friendship), the role of the family in choosing a spouse, and having a relationship with another person during marital life on the marriage survival have been modeled using survival analysis in the presence of competing risks.

## Methods

### Study design and population

This longitudinal study was conducted on 386 couples who were selected by random quota sampling and were married from 1991 to 2018. The participants’ marriage was registered in the general register office in Tabriz, Iran. At this time, individuals who experienced a divorce took into account the event of interest, and those who continued to live together were regarded as censored.

### Ethical consideration

The proposal of this study was approved by the intuitional review board of Tabriz University of medical sciences (Ethics code: IR.TBZMED.REC.1397.668). All participants were informed of the objective and design of the study and the confidentiality of their personal information. Written consent was received from all participants, and they were free to leave the study whenever they wish. The required scientific principles were regarded according to strengthening the reporting of observational studies in epidemiology (STROBE) statement.

### Statistical analysis

Data were analyzed using STATA software version 15 (Chicago, IL., USA). Data were expressed using mean (standard deviation; SD) and frequency (%) for numeric and categorical variables, respectively. In the next step, the causes of moral-religious commitment, how to get acquainted with each other (traditional or friendship), the role of the family in choosing a spouse and being in a relationship with someone else were considered as competing risks and were modeled using Lunn-McNeil approach [15]. Instead of considering a separate model for each of the events, the Lunn-McNeil approach fits a general model. It also does not consider competing risks as censorship. Significant factors (P <0.05) from univariate analysis were candidates as to inter in the multivariate analysis. Based on the selected model and for each of the factors included in the model, the cause-specific Hazard Ratios (HRs) (and their 95% CI) were reported as the effect size of interest. In the multivariate step, a p-value less than 0.05 was considered significant.

## Result

The number of participants in this study was 386. A total of 54.7% of the participants were female and 45.4% were male. The average age of marriage in individuals was about 23.9 (SD 6.6) years, and in their spouses was about 25.2 (SD 6.9) years. The average age difference between couples was about 5.3 (7 to 18) years.

According to the words of the participants, 47.9% had a dowry of fewer than 100 coins and 13.2% of them had a dowry of more than 400 coins. About 30.7% of the participants had a university education and about 40.9% of the participants’ spouses had a university education. The level of education in about 68.6% of the participants was either not different from their spouse or was lower than that. Of course, reports indicate that this similarity was greater than that before marriage (about 74.9%).

A total of 54.8% of the participants were in the category of professional-technical occupations, 1.6% were in the category of occupations related to administrative and secretarial affairs, 1.9% were related to transportation and 2.2% were in the category of jobs related to military services. Spouses of most of the participants in the study (42.9%) were in the category of professional-technical occupations and a low percentage of them were in the category of occupations related to administrative and secretarial affairs (1.5%), occupations related to transportation were 2.1% and the category of jobs related to military services was 2.4%.

Regarding the couple’s residence after marriage, about half of those surveyed said they lived far from their families. The participants stated that more than half of them had the same place of residence, more than 99% of them had the same nationality, about 95% of them had the same race, and more than 75% of them had the same place of birth. More than 91% of the participants with their spouses were physically healthy and more than 62% of the participants with their spouses had job stability before and after marriage. More than 70% of the participants with their spouses had income stability before and after marriage. About 60% of the individuals in the study had one child, 7.9% of them had 3 or more children, and about 65% of couples had an engagement period of less than 1 year (Tables 1 and 2).

**Table 1:** Demographic profile of study participants.

Variables	Frequency	Percentage
**Gender**		
Male	172	45.3
Female	208	54.7
**The amount of dowry**		
Under 100 gold coins	171	47.9
Between 100 and 200 gold coins	77	21.6
Between 200 and 300 gold coins	31	8.7
Between 300 and 400 gold coins	31	8.7
Over 400 gold coins	47	13.2
**Level of education**		
illiterate	7	1.9
Partly illiterate	6	1.6
Primary school	31	8.2
Guidance school	42	11.1
High school	37	9.8
Diploma	139	36.8
Associate Degree	34	9.0
B.Sc.	72	19.0
M.Sc.	7	1.9
Ph.D.	3	.8
**Spouse’s education level**		
illiterate	7	1.9
Partly illiterate	4	1.1
Primary school	28	7.4
Guidance school	43	11.4
High school	25	6.6
Diploma	115	30.6
Associate Degree	41	10.9
B.Sc.	90	23.9
M.Sc.	11	2.9
Ph.D.	12	3.2
**Matching the level of education of couples**		
No difference	146	38.8
Slight difference	112	29.8
There is a large difference	70	18.6
Too much difference	48	12.8
**Matching the level of education of couples before marriage**		
No difference	150	39.7
Slight difference	133	35.2
There is a large difference	67	17.7
Too much difference	28	7.4
**Job-status**		
professional-technical occupations	171	54.8
jobs related to administrative affairs and secretarial affairs	5	1.6
Sales Jobs Category	13	4.2
Transportation-related jobs	6	1.9
Industrial professions and production operations	38	12.2
Services related to performing services (services)	72	23.1
Job-related to military services	7	2.2
**Occupational status of spouse**		
Professional-technical occupations	142	42.9
Job-related to administrative affairs and secretarial affairs	5	1.5
Sales Jobs Category	13	3.9
Transportation-related jobs	7	2.1
Industrial professions and production operations	38	11.5
Services related to services	118	35.6
Job-related to military services	8	2.4
**Place of residence after marriage**		
We lived far from our families	175	47.3
We were with the wife’s family (under the same roof)	71	19.2
We were in my wife’s family neighborhood	64	17.3
We were in my family’s neighborhood	41	11.1
My family and I lived under one roof	19	5.1
**Homogeneity of the couple’s place of residence**		
No difference	174	51.6
There is a difference	163	48.4
**Matching the couple’s nationality**		
No difference	382	99.7
There is a little difference	1	.3
**Matching the couple’s race**		
No difference	361	94.8
There is a difference	20	5.2
**Matching the couple’s place of birth**		
No difference	260	75.6
There is a little difference	84	24.4
**The physical condition of the couples**		
Both health	345	91.3
One of them is sick	29	7.7
Both of them are sick	4	1.1
**Couples’ job stability before and after marriage**		
Both were stable	186	62.8
One of the couples was stable	95	32.1
None of them were stable	15	4.8
**Couples’ income stability before and after marriage**		
Both were stable	249	70.7
One of the couples was stable	85	24.1
None of them were stable	18	5.1
**Number of children**		
1	166	59.7
2	90	32.4
3+	22	7.9
**Time being engaged**		
Less than six months	122	31.9
Six months to 12 months	125	32.6
12 months to 18 months	72	18.8
18 months to 24 months	25	6.5
More than 24 months	39	10.2
**Number of divorces**	120	31.1

**Table 2:** Competing risk information.

Variables	Frequency	Percentage
**Having a relationship with another person**		
Yes	80	21.7
No	289	78.3
**How to get acquainted with your spouse**		
We met by parents (traditional method)	212	57.8
New methods (such as friendship or the Internet) or by force	154	42.2
**The role of the family in choosing a spouse**		
Very low—medium	442	57.3
Too much and too much	330	42.7
**The role of the moral-religious status of the spouse**		
Very low—medium	312	40.4
Too much and too much	460	59.6

### Competing risks

In this part of the study, the causes and factors related to marriage survival were investigated separately. About 22% of the participants stated that their spouse was in a relationship with someone else. About 58% of the participants got familiar with their spouses through traditional methods, and the rest were introduced and married by new methods (such as friendship or the Internet) or by force. The role of the family in choosing a spouse was very low to moderate for about 57% of the participants. The role of the moral-religious commitment of the spouse in choosing a spouse was high and very high for about 60% of the people in the study.

The average time to divorce was 109 days, which ranged from 6 to 267 days. In this time range, the number of divorces was 120 of 386 cases (31.1%) of couples in the study.

### Having a relationship with another person

According to the results of the Lan-McNeil model, there was a direct and significant relationship between the risk of divorce and the same place of residence before marriage, illness of one of the spouses, and having more than 2 children. Also in this analysis, the interaction of competing risk of having a relationship with another person with a low dowry, the same place of residence before marriage, and illness of both couples were positive and significant, which indicates having a relationship with another person along with low dowry, the same place of the residence before marriage, the illness of both couples increased the risk of divorce. Based on the results of the Lunn-McNeill multivariate analysis, there was a direct and significant relationship between the risk of divorce and the difference in the education level between couples before marriage, the illness of one couple, and having more than 2 children (Table 3).

**Table 3:** Results of Lunn-McNeil multivariate analysis for the competing cause of having a relationship with another person.

Variables	HR	L	U	P-value
**Number of children 2+**	.27	.09	.78	**0.016**
The interaction of competing causes with the number of children 2+	1.12	.25	5.05	0.879
**Matching the education of couples before marriage**				
Slight difference	.23	.06	.80	**0.021**
Large difference	.88	.25	3.10	0.843
Very large difference	.15	.03	.70	**0.015**
Interaction of competing causes with a slight difference	2.03	.42	9.83	0.379
Interaction of competing causes with large differences	1.61	.35	7.27	0.539
Interaction of competing causes with very large differences	2.25e-15	---	---	1.000
**Physical condition**				
One of the sick couple	18.06	5.38	60.65	**<0.001**
Both patients	187.63	13.61	2586.81	**<0.001**
Interaction of competing causes with one of the sick couples	8.85e-17	---	---	1.000
Interaction of competing causes with both patients	.002	---	---	1.000

LR Chi2(11) = 46.28, Log likelihood = -168.87485, P-Value< 0.0001.

HR: Hazard Ratio: Lower bound of 95% confidence Interval; U: Upper bound of 95% Confidence Interval.

### Being familiar with the spouse traditionally

The results of a Lunn-McNeil analysis showed that there was a direct and significant relationship between the risk of divorce and having a difference in the education level of the couple and also a large difference in the education level of the couple before marriage, illness of one of the couples, having more than 2 children and more than 7years age difference. Also in this analysis, the interaction of competing risk of being familiar with the spouse traditionally with a large difference in education level and illness of both couples has been positive and significant, which shows how a traditional way of meeting a spouse along with a large difference in the level of education and illness of both couples increased the risk of divorce. Based on the results of the Lunn-McNeill multivariate analysis, there was a direct and significant relationship between the risk of divorce and having a large difference in the education level before marriage, the illness of one spouse, and having more than 2 children (Table 4).

**Table 4:** Results of Lunn-McNeil multivariate analysis for the competing cause of traditional mode of being familiar with spouse.

Variables	HR	L	U	P-value
**Number of children 2+**	.14	.03	.62	**0.010**
The interaction of competing causes with the number of children 2+	3.39	.62	18.45	0.158
**Matching the level of education of couples before marriage**				
Slight difference	.46	.14	1.52	0.206
Large difference	.77	.17	3.51	0.740
Very large difference	.18	.04	.66	**0.010**
Interaction of competing causes with a slight difference	.41	.08	2.11	0.285
Interaction of competing causes with large differences	1.55	.28	8.55	0.615
**Physical condition**				
One of the sick couple	9.60	2.43	37.85	**0.001**
Both patients	.59	.10	3.32	0.546
Interaction of competing causes with one of the sick couples	.86	.23	3.16	0.820
Interaction of competing causes with both patients	3.11	.69	13.95	0.139

LR Chi2(11) = 36.47, Log likelihood = -173.78092, P-Value = 0.0001.

HR: Hazard Ratio: Lower bound of 95% confidence Interval; U: Upper bound of 95% Confidence Interval.

### Role of the family in choosing a spouse

According to the results of the Lunn-McNeill, there was a direct and significant relationship between the risk of divorce outcome with a very large difference in the education level of the couples and also a large difference in the education level of the couples before marriage, illness of one or both couples, having more than 2 children and more than 7 years age difference. Also in this analysis, the interaction of the insignificant role of the family in choosing a spouse with a dowry of more than 400 coins, the large difference in the level of education of couples, and the lack of job stability of both couples have been positive and significant, which shows the insignificant role of the family in choosing a spouse with a dowry over 400 coins, the large difference in the education level of the couples and the lack of job stability of both couples reduces the survival of the marriage. Based on the results of the Lunn-McNeill multivariate analysis, there was a direct and significant relationship between the risk of divorce outcome with the illness of one or both spouses, differences in the level of education before marriage, the similarity of the place of birth and having more than 2 children (Table 5).

**Table 5:** Results of Lunn-McNeil multivariate analysis for the competing cause of family weak role in choosing a spouse.

Variables	HR	L	U	P-value
**Physical condition**				
One of the sick couple	16.55	5.36	51.04	**<0.001**
Both patients	82.48	6.13	1109.86	**0.001**
**Matching the level of education of couples before marriage**				
Slight difference	.21	.06	.68	**0.009**
Large difference	1.53	.60	3.91	0.373
Very large difference	.086	.017	.42	**0.003**
Interaction of competing causes with the slight difference	2.49	.46	13.54	0.291
Interaction of competing causes with large differences	.28	.031	2.43	0.245
Interaction of competing causes with very large differences	3.25	.22	47.18	0.388
**Number of children 2+**	.39	.16	.94	**0.036**
The interaction of competing causes with the number of children 2+	.26	.03	2.43	0.240
**Matching the couple’s birthplace**	2.45	1.04	5.81	**0.041**
Interaction of competing causes with a similar place of birth	.249	.04	1.60	0.143

LR Chi2 (10) = 43.78, Log likelihood = -170.12572, P-Value<0.0001.

HR: Hazard Ratio: Lower bound of 95% confidence Interval; U: Upper bound of 95% Confidence Interval.

### Moral-religious commitment in choosing a spouse

According to a Lunn-McNeill model, there was a direct and significant relationship between the risk of divorce and having a very large difference in the education level of the couples and also a large difference in the education level of the couples before marriage, illness of one or both couples and having more than 2 children. Also in this analysis, the interaction of competing risk of having a low role of moral-religious commitment in choosing a spouse with similarity of nationality, having the same race, and lack of income stability of one of the couples was positive and significant, which shows the low role of moral-religious commitment in choice with the lack of income stability of one of the couples, reduces the survival of the marriage. Based on the results of the Lunn-McNeill multivariate analysis, there were direct and significant relationships between the risk of divorce and the illness of one or both couples, the difference in education level before marriage, and having more than 2 children (Table 6).

**Table 6:** Results of Lunn-McNeil multivariate analysis for the competing cause of the role of moral- religious commitment in choosing a spouse.

Variables	HR	L	U	P-value
**Physical condition**				
One of the sick couple	8.89	3.09	25.59	**<0.001**
Both patients	112.64	8.86	1432.02	**<0.001**
**Matching the level of education of couples before marriage**				
Slight difference	.42	.15	1.16	0.096
Large difference	1.56	.589	4.11	0.372
Very large difference	.18	.04	.82	**0.027**
Interaction of competing causes with the slight difference	.47	.09	2.46	0.369
Interaction of competing causes with large differences	.42	.08	2.23	0.311
Interaction of competing causes with very large differences	.38	.04	3.74	0.410
**Number of children 2+**	.37	.15	.90	**0.029**
The interaction of competing causes with the number of children 2+	.43	.083	2.29	0.326

LR Chi2 (10) = 39.82, Log likelihood = -172.10594, P-Value< 0.0001.

HR: Hazard Ratio: Lower bound of 95% confidence Interval; U: Upper bound of 95% Confidence Interval.

## Discussion

Studies used to model competing risk data so far mainly used standard methods of survival analysis such as Cox regression. Censorship of competing risks and ignoring risks is the most critical challenge to the Cox model in analyzing competing risk data. Because it may lead to incorrect estimates, the Cox regression analysis’s validity also depends heavily on the proportional hazards (PH) assumption. If the assumption is not met of independence, this model is not as classically suitable for competing for risk setting.

The results of the present study, which examined four competing risks, indicated that only one factor did not reduce the survival of the couple. Rather, having a relationship with another person, traditional marriage, the role of moral-religious commitment, insignificance role of the family in choosing a spouse along with other cause increases the risk of marriage survival reduction.

Problems leading to divorce could be present from the beginning of the relationship or may arise and worsen during the marriage [16]. Similar to our findings, other studies found that one of the most commonly reported causes was infidelity which led to various mental problems, followed by growing apart and finally divorce [17–20]. Couples’ therapists believe infidelity is one of the most detrimental events in the relationship and spiritual well-being can be helpful in divorce caused by infidelity [21].

In this study, the role of morality and religious attitude among competing risks was obtained as the first factor (59.6%). The results of previous studies show that considering the religious beliefs of the spouse before marriage can ensure higher marital quality, which is consistent with previous studies [22–24]. As well as pre-marriage moral values are important and fundamental factors for making marriage survival [25] and religiosity can reduce the risk of infidelity as well as divorce [26].

As one of the results, traditional marriage (57.8%) along with a large difference in the level of education and illness of both couples, is another major cause of reducing the survival of the marriage, which is consistent with the results of another study [27]. Therefore, informing people to reduce traditional marriage with increase prior knowledge of couples about each other can be one of the main strategies for preventing divorce in Islamic countries such as Iran [28]. According to the National Fatherhood Initiative report, unrealistic expectations, infidelity, lack of commitment in the relationship, traditional marriage and violence are the main causes of divorce in the US [29]. In our study high amount of dowry which can be considered an unrealistic expectation, has increased the divorce rate.

The educational difference in many studies has high divorce risk because of encountering better alternatives for marriage [30]. We found the educational difference between the couple and having illness in the couples are other causes of divorce. Other studies indicate that chronic problems powerfully affect the marital relationship and can lead to dissatisfaction and finally divorce [31,32]. Another issue is financial problems that in addition to leading to divorce, can also be a problematic consequence of divorce, especially for women [19]. One of the best strategies for reducing the psychological consequences of divorce can be social support [33].

To our knowledge, no study has yet used this model to identify competing risks affecting marriage survival. Considering the importance of the issue of marriage survival and the fact that different factors cause divorce in different societies, we had limited our study to one approach. It is suggested that different approaches be used for this issue in future studies. We implicitly assumed that the study population was homogeneous and that all participants were at equal risk. But some unmeasured risk factors affect the risk function. It is suggested that the frailty model be used in future studies to investigate the effects of such factors.

## Conclusion

The results of the study showed that various factors were effective in reducing marriage survival, and it is not possible to determine just one major and definitive cause of divorce. Therefore, recognizing these factors is important in understanding marriage survival, because the role and importance of factors in different societies and cultures are different. This knowledge allows timely intervention to increase the survival of marriage for each couple, their family, psychologists, and social workers at the micro level and for sociologists and policymakers at the macro level.
